# Roles of Antimicrobial Peptides in Gynecological Cancers

**DOI:** 10.3390/ijms231710104

**Published:** 2022-09-03

**Authors:** Chongyi Zhao, Shuo Yan, Yuzhu Song, Xueshan Xia

**Affiliations:** Faculty of Life Science and Technology, Kunming University of Science and Technology, Kunming 650500, China

**Keywords:** antimicrobial peptide, gynecological cancers, anticancer peptide, biomarker, tumorigenic

## Abstract

Antimicrobial peptides (AMPs) are essential components of the mucosal barrier of the female reproductive tract (FRT) and are involved in many important physiological processes, including shaping the microbiota and maintaining normal reproduction and pregnancy. Gynecological cancers seriously threaten women’s health and bring a heavy burden to society so that new strategies are needed to deal with these diseases. Recent studies have suggested that AMPs also have a complex yet intriguing relationship with gynecological cancers. The expression level of AMPs changes during tumor progression and they may act as promising biomarkers in cancer detection and prognosis prediction. Although AMPs have long been considered as host protective, they actually play a “double-edged sword” role in gynecological cancers, either tumorigenic or antitumor, depending on factors such as AMP and cancer types, as well as AMP concentrations. Moreover, AMPs are associated with chemoresistance and regulation of AMPs’ expression may alter sensitivity of cancer cells to chemotherapy. However, more work is needed, especially on the identification of molecular mechanisms of AMPs in the FRT, as well as the clinical application of these AMPs in detection, diagnosis and treatment of gynecological malignancies.

## 1. Introduction

Antimicrobial peptides (AMPs) represent ancient host defense molecules present in all life forms [1,2]. The vast majority are cationic peptides which can directly target negatively charged surfaces of specific organisms. Anionic AMPs are rare and they may act by using metal ions to form cationic salt bridges with negatively charged components of microbial membranes [3]. Besides, AMPs may exert antimicrobial effects through intracellular targeting such as binding to the nucleic acid and proteins, affecting cell cycles and disrupting energy metabolism [4]. In addition to the microbicidal and anti-inflammatory functions, AMPs also have immunomodulatory properties including enhancing chemotaxis of immune cells, activating immune cell differentiation, stimulating angiogenesis, improving wound healing and reducing scar formation [5,6].

Recently, the relationship of AMPs and cancers has attracted extensive attention of researchers [6,7]. The expression of AMPs is altered in tumors [8,9], which may serve as biomarkers for detecting tumors at an early stage [10]. Several studies have found that AMPs exhibit tumorigenic effects, such as conferring resistance to apoptosis in tumor cells [11], stimulating tumor migration [12,13], enhancing angiogenesis and promoting lymphatic invasions [14,15]. AMPs also act as chemotactic factors that recruit monocytes, immature dendritic cells, memory T cells, mast cells and tumor-associated macrophages (TAMs) to cancerous lesions, thereby altering the tumor environment and promoting tumor progression [16,17,18]. However, many studies indicated that AMPs exert antitumor effects. AMPs kill cancer cells through electrostatic interactions [19] or by activating necrosis or apoptosis through various signaling pathways [20,21]. AMPs also inhibit tumorigenesis by activating the immune system [22], inhibiting DNA synthesis [23], and reducing angiogenesis [24]. Moreover, as some cancers are induced by specific organisms [25,26], a complex relationship may exist between AMPs, local microbiota, and malignancy. 

Gynecological cancers, mainly including ovarian, uterine, cervical, vaginal and vulvar, place a heavy burden on society and patients. It was reported that in 2020, there were 1,398,601 new cases and 671,875 deaths from gynecological cancers worldwide. While the incidence and mortality rate of cervical cancer is decreasing rapidly in high-income countries due to human papillary virus (HPV) vaccination and screening, the incidence rate of uterine corpus cancer is increasing in some of these areas because of body overweight and lack of exercise. According to the growth and aging of the population, by the year of 2040, the total global cancer burden is expected to be 28.4 million new cases [27]. Gynecological cancer patients often experience severe anxiety and depression, as well as poor quality of life [28]. To reduce morbidity and mortality, studies are needed to improve or complement existing screening, diagnosis and treatment strategies and to further explore the pathogenesis. The association between AMPs and gynecological cancers has received much attention in recent 15 years, but has not been well summarized. In this review, we provide a critical analysis of the relevant literature to underpin a better understanding of the role of female reproductive tract (FRT)-derived AMPs in gynecological malignancies and elucidate the potential application of AMPs in these diseases.

## 2. Antimicrobial Peptides in the Female Reproductive Tract (FRT)

AMPs have been found in different human excretions, tissues and cell types. Likewise, a set of AMPs have been identified in the FRT. These AMPs not only function as primary barrier against pathogen invasion, but also help shape the microbiota composition [29]. During evolution, commensals have developed resistance strategies against host-derived AMPs, secreted their own AMPs to compete with potential pathogens, and finally survived as symbionts [30]. In addition, AMPs are important in dampening inflammation and maintaining immune homeostasis in the FRT [29]. During pregnancy, AMPs are distributed throughout the FRT and the antimicrobial, anti-inflammatory and immunoregulatory activities of the AMPs are essential in protecting and maintaining pregnancy. It has been demonstrated that abnormal expression of AMPs is associated with ectopic pregnancy, preterm labor, intra-amniotic infection/inflammation, premature rupture of membranes, and cervical insufficiency [31,32]. The activities of AMPs are influenced by the proteases, protease inhibitors, pH and hormonal changes [33] and AMPs usually display synergistic effects rather than work individually [34]. However, a large number of AMPs in the FRT remain to be discovered. For this reason, several studies have used proteomes and peptidomes to reveal potential AMPs in human cervical-vaginal fluid and endometrial fluid in order to complement the composition as well as functions of AMPs in FRT [35,36,37,38].

We have summarized the general information on AMPs associated with gynecological cancers in Table 1. All of these tumor-related AMPs exert antimicrobial and immunoregulatory functions and some of them have other specific activities. For example, secretory leukocyte protease inhibitor (SLPI) and elafin confer protease inhibition activity which plays an important role in tissue integrity [39] and low expression of elafin is associated with pelvic organ prolapse and urinary stress incontinence [40,41].

## 3. Expression of AMPs in Gynecological Cancers

Through a literature review, we found that the expression of AMPs is altered in gynecologic cancers and details are shown in Table 2. In these studies, protein or/and mRNA expression was evaluated in different samples including patient-derived tumor samples, cancer cell lines, xenograft tumor tissues, serum, circulating tumor cells and cystic fluid. It can be found that the expression of a certain AMP can be elevated or decreased in different types of tumors, indicating that the expression of AMP depends on the tumor type and is not specific. Moreover, even in the same tumor type, AMP expression varies by specimen types. For example, HBD3 protein, which is highly expressed in cervical cancer tissues, is not expressed in cervical cancer cell lines [12]. This inconsistency may suggest that the signaling and metabolic pathways in transformed cell lines differ markedly from tumor tissues, whose microenvironment may be involved in the regulation of AMP expression. Therefore, studies on cell lines cannot fully represent the actual situation of the tumors. More realistic studies based on the tumor itself can reveal more about the role of AMPs. Meanwhile, the AMP expression levels are found to be correlated with tumor progression [12,55,56,57,58,59,60], clinical stages [59,61,62,63,64,65], tumor grading [55,66,67,68], lymph node metastasis [58,68,69] and even the amount of ascites [69]. Prognostic indicators such as progression-free survival (PFS) and overall survival (OS), were also found to be associated with AMP expression [61,62,63,64,70,71,72]. In addition, AMPs, alone or in combination with other molecules, are potential biomarkers in indicating malignancies, predicting early cancers and monitoring recurrence [61,62,63,73,74,75,76,77,78,79,80,81]. Particularly and interestingly, when expressed in nuclei and cytoplasm, elafin is a biomarker of more aggressive cervical cancer. In contrast, elafin expression in cell membrane indicates a more conservative cervical cancer [57].

AMP expression in gynecological cancers is tumor-type specific and correlates with various clinical features and outcomes. However, the mechanism by which AMP expression changes in gynecological cancers is unclear. Only one study mentioned that the overexpression of elafin in ovarian cancers is mediated by inflammatory cytokines through nuclear factor kappa B (NF-κB) pathway [70]. Moreover, whether altered AMP expression is a cause or a consequence of tumors is currently unknown. Nevertheless, AMPs are promising biomarkers for identifying cancers, indicating aggressiveness and assessing prognosis. 

## 4. Tumorigenic and Pro-Metastatic Functions of AMPs

**Figure 1:**
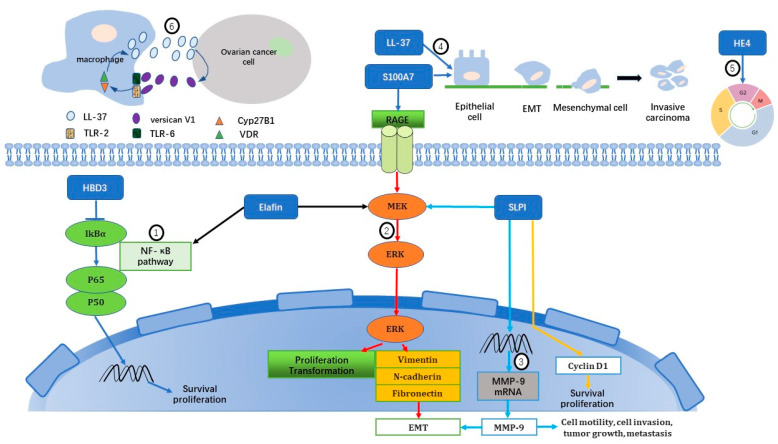
Tumorigenic and pro-metastatic mechanisms of AMPs on gynecological cancers: (1) HBD3 and elafin activate NF-κB pathway [12,95]; (2) Elafin, S100A7 and SLPI induce MEK/ERK pathway [58,68,87,95,96]; (3) SLPI promotes extracellular matrix remodeling and angiogenesis [68,97]; (4) LL-37 and S100A7 induce epithelial-mesenchymal transition (EMT) [58,98]; (5) HE4 controls cell cycle [91]; (6) A vicious circle between macrophages and ovarian cancer cells by interaction between LL-37 and versican V1 [99]. MEK, Mitogen-activated protein kinases; ERK, Extracellular signal-regulated kinases; RAGE, Receptor for advanced glycation end-products; EMT, Epithelial–mesenchymal transition; VDR, vitamin D receptor; TLR, Toll-like receptor; MMP, Matrix metalloproteinases; CYP27B1, Cytochrome P450 family 27 subfamily B member 1.

To turn normal cells into cancer cells, genes that regulate cell growth and differentiation must be altered and several signaling pathways are involved. Recent comprehensive studies in the Cancer Genome Atlas (TCGA) have revealed that there are twelve driver events per tumor [92], and there is considerable variation in the genes and signaling pathways altered across different tumor types and individual samples [93]. Therefore, knowledge of the molecular subtype is essential in cancer management. For example, molecular classification is now encouraged as clinical routine and as determinants for treatment decisions in uterine endometrial cancer. As for metastasis, there are currently three theories that explain the mechanisms [94]: (1) the epithelial-mesenchymal transition (EMT) and mesenchymal-epithelial transition (MET) hypothesis, (2) the cancer stem cell hypothesis, and (3) the macrophage–cancer cell fusion hybrid hypothesis. Likewise, in gynecological cancers, AMPs exert tumorigenic activities through diverse oncogenic signaling pathways and promote metastasis via EMT and macrophage participation. The mechanisms have been summarized and shown in Figure 1. In addition, copy number variation (CNV) and distribution polymorphisms of the AMP gene are also associated with tumorigenesis. 

HBD3 enhances proliferation and migration of cervical cancer cells both in vitro and in vivo by activating the NF-κB signaling pathway [12]. NF-κB is known to not only activate the genes that keep the cell proliferating and protect the cell from apoptosis, but also lead to metastasis and inefficient eradication of the tumor by the immune system.

S100A7 promotes the migration, invasion and metastasis of cervical cancer cells via activating extracellular signal-regulated kinases (ERK) pathway both in vitro and in vivo. Moreover, S100A7 enhances cell mesenchymal properties and induces EMT, which enables cancer cells to invade surrounding tissues and disseminate to distant organs, resulting in invasion and metastasis [58]. Knocking down of S100A7 reduces the ability of proliferation, migration, and invasion of ovarian cancer cells [68].

LL-37 contributes to ovarian tumorigenesis through stimulation of tumor cell growth, angiogenesis and recruitment of immune cells [87]. LL-37 may activate mitogen-activated protein kinase (MAPK) and Janus-activated kinase/signal transducers and activators of transcription (JAK-STAT) signaling cascades in ovarian cancer cells, upregulate a number of transcription factors related to tumorigenesis such as CREB and STAT4, and regulate the expression of several genes associated with tumor progression, such as *egf*, *mmp2* and *upa* [98]. Studies have shown that mesenchymal stem cells (MSCs) contribute to progression and metastasis of ovarian cancers [100]. LL-37 may mediate MSCs migration and invasion through formyl peptide receptor like-1(FPRL1) and MSCs exposure to LL-37 leads to secretion of angiogenic and inflammatory molecules such as interleukin (IL)-1 receptor antagonist, IL-6, IL-10, vascular endothelial growth factor (VEGF) and matrix metalloproteinase-2 (MMP-2), all of which are associated closely with tumorigenesis and metastasis [101]. Moreover, neutralization of LL-37 reduces ovarian tumor growth by decreasing recruitment of MSCs in a xenograft mouse model [101]. Macrophages are also shown to be associated with tumor growth. A co-culture model, containing macrophages and ovarian cancer cells, demonstrates that versican V1, a chondroitin sulfate proteoglycan produced by ovarian cancer cells, can induce hCAP18/LL-37 overexpression in macrophages through activation of toll-like receptor 2 (TLR2) and TLR6 and subsequent vitamin D-dependent mechanisms, leading to proliferation and invasiveness of ovarian cancer cells. Meanwhile, proliferation of ovarian cancer cells leads to elevated expression of versican V1 [99]. As a result, a vicious circle is formed. We have noted that in these studies, the concentrations of LL-37 are between 0.1 μg/mL and 10 μg/mL, which show tumorigenic effects, while in another study described later in this review, LL-37 at a concentration of 50 μg/mL helped increase cell membrane permeability, so that the CpG-oligodeoxynucleotides (CpG-ODN) could easily enter the cell to exert subsequent effects [102]. This demonstrates that the effects of LL-37 are concentration-dependent.

SLPI promotes ovarian cancer cell growth, prevents apoptosis in vitro and exerts a pro-metastatic function via increasing MMP-9 production in vivo [97]. SLPI protects the survival factor progranulin, partly through inhibition of elastase-induced degradation or independently of protease inhibition [103,104]. However, a quite opposite result shows that SLPI inhibits cell proliferation, increases apoptosis and decreases the invasive ability of ovarian cancer cells in vitro through tumor necrosis factor (TNF)-related apoptosis-inducing ligand (TRAIL), death receptor (DR)-4, DR-5, TNF-α, and TNF receptor (TNFR)-I expression, all of which may lead to activation of the apoptosis pathway through Caspase-2, Caspase-8 and Caspase-9 [69]. The reasons for the conflicting results are not clear, but may be due to the different cancer cell lines the study chose and the data obtained in vitro or in vivo. In uterine neoplasm, SLPI activates proliferation of endometrial adenocarcinoma cells directly through its control of *cyclin D1* gene expression. Meanwhile, SLPI inhibits the expression of the growth suppressors insulin-like growth factor-binding protein 3(IGFBP-3) and transforming growth factor beta 1(TGF-β1) in an indirect pathway. Furthermore, SLPI negatively regulates lysyl oxidase (a tumor suppressor) gene expression. The results of these multiple regulations are the synergistic induction of cancer cell proliferation [105].

HE4 is currently used as a biomarker for ovarian and endometrial cancer, but its relationship with tumors is unclear. HE4 is shown to enhance endometrial cancer cell proliferation, both in vitro and in vivo, possibly by cell cycle control. HE4 also promotes invasion and metastasis although the mechanism is not clear [91].

TCGA data has shown that somatic copy number alterations (SCNAs) are pervasive across cancers although the exact relationship between SCNAs and cancers is largely unknown. Copy number variation (CNV) in the DEFB4 gene (encoding HBD4) exists in both cervical cancer and healthy control groups, and a lower DEFB4 copy number is possibly associated with susceptibility to cervical cancers [106]. In addition, lower copy number of DEFB4 is also associated with susceptibility to human immunodeficiency virus infection (HIV) [107] and decreased antimicrobial activity [108]. Since the majority of the cervical cancers are HPV-related, whether lower copy number of DEFB4 also contributes to susceptibility to HPV infections is an open question.

It is known that a polymorphic variant of a gene can lead to the abnormal expression or to the production of an abnormal form of the protein, which may cause or be associated with disease. The distribution of lactoferrin gene polymorphisms (rs1126477, rs1126478, rs2073495, and rs9110) was investigated and it was shown that rs1126477 was significantly associated with ovarian cancer in the Chinese Han population. In addition, the frequency of the A allele of rs1126477 was significantly higher in ovarian cancer patients than in controls. Therefore, rs1126477 may play a role in physiological processes of ovarian cancers in the Chinese [109].

The carcinogenic mechanisms of AMPs are complex and vary by tumors, research subjects, AMP concentrations and in vitro or in vivo studies. In addition, for anticancer purposes, whether neutralization of AMPs or anti-AMPs strategies can be used as cancer therapy is still a concern for researchers.

## 5. Anti-Tumor Functions of AMPs

The antitumor property of AMPs is mainly based upon selective binding to cancer cells via electrostatic interactions. Most AMPs are cationic peptides that specifically target negatively charged cell membranes. Cancer cell membranes typically carry a net negative charge due to a higher expression of anionic molecules such as phosphatidylserine and O-glycosylated mucins compared to normal cells [110,111]. However, the density of negative charge on cancer cells is relatively lower when compared to bacterial cell membranes. Consequently, the affinity of AMPs to cancer cells is between that of normal cells and bacteria [112]. In addition, cancer cells contain less cholesterol and more microvilli than normal cells, which makes them more susceptible to killing and binding by AMPs [113,114]. Therefore, the cell membrane composition, fluidity, and surface area in different cancer cells may account for the selective killing efficacy observed in different AMPs.

HBD-2 kills Hela cells through acute lytic cell death [115]. The cell-killing effect of HBD-2 is concentration-dependent, with low concentrations promoting proliferation and high concentrations causing death [55]. Unfortunately, when compared to other defensins, HBD-2 requires much higher concentrations to kill tumor cells due to its relatively lower net positive charge [115]. However, higher concentrations may exert higher toxicity and kill normal cells as well. Furthermore, the action of HBD-2 could be severely compromised in serum [116], making intravenous delivery of this potential drug problematic. HBD-2 is more effective when delivered directly into tumor cells [117] although successful and safe delivery is still a challenge. However, recent studies have made progress in delivery of AMPs by conjugating with nano carriers [118], which brings AMP targeting therapy a promising future.

In addition to cell membrane lytic function, AMPs can penetrate cancer cells and attack the mitochondria, leading to apoptosis [119]. A recombinant fragment of human SP-D (rfhSP-D) decreases the motility and proliferation of ovarian cancer cells by inhibiting the mammalian target of rapamycin (mTOR) activity, increasing caspase 3 cleavage, and inducing pro-apoptotic genes *Fas* and *TNF-α* [120]. When rfhSP-D was immobilized on carbon nanotubes (CNTs) and added to culture system of ovarian cancer cells, apoptosis of the cells was induced [121].

Currently, while vaccines can greatly prevent some HPV infections, vaccination rates show geographic disparities, so that HPV prevalence remains high in some regions [122]. Therefore, preventing or blocking HPV infection remains important in terms of cervical cancer prevention. HD5 has been shown to potently prevent infection from multiple serotypes of HPV, including HPV16 [123,124,125,126], which, together with HPV18, accounts for about 70% of all the cervical cancers [127]. HD5 binds HPV capsid outside the host cell and the binding virus is internalized into the cell. After that, HD5 prevents dissociation of the viral capsid from the genome, reduces viral trafficking to the trans-Golgi network, leads the viral particle to the lysosome, and accelerates the degradation of internalized capsid proteins [128,129]. Likewise, HD5 has the same effect on adenoviruses [130], which seems to suggest that HD5 has the same effect on non-enveloped viruses. Although HD5 only reduces the likelihood of HPV infection, rather than completely blocking the process, it is still effective in preventing HPV-related cervical cancer.

Under normal conditions, the immune system is involved in identifying and killing cancer cells, while during tumorigenesis, immunosurveillance occasionally fails and cancer grows [131]. Dendritic cells (DC) play an important role in cervical immunity and have been shown to be deficient in cervical cancer [132,133]. Human neutrophil peptide 2(HNP2) can recruit DC in organotypic cultures of HPV-transformed keratinocytes maintained in vitro or grafted in vivo and then restore the immune functions altered by DC deficiency [89]. It is suggested that DC vaccination may activate the adaptive immune system to detect and eliminate the cancer cells [134]. Therefore, restoring the immune balance altered in cancers may be a promising option for fighting cancers.

AMPs can also be combined with other molecules to enhance anticancer effects. The combination of LL-37 and CpG-oligodeoxynucleotides (CpG-ODN), a toll-like receptor (TLR9) ligand, increases the delivery of CpG-ODN into endosomes and elevates interferon γ (INFγ) expression. Consequently, this process induces proliferation and activation of NK cells and subsequently inhibits the cancer cells in murine ovarian cancer models [102]. In this study, LL-37 is used to provide synergistic molecules to increase the uptake of CpG-ODN into immune cells and thus enhance antitumor effects. The study demonstrates that at higher concentrations (50 μg/mL and 100 μg/mL), LL-37 makes the cell membrane more permeable, allowing other molecules to enter the cell more easily to perform their functions.

By regulating cyclinD1, MMP9 and p27, knockdown of S100A7 reduces epithelial ovarian cancer (EOC) cell proliferation, migration and invasion, and enhances chemosensitivity to cisplatin. It is shown that microRNA(miRNAs) can be used as biomarkers of early detection of cancers, as well as tools or targets for treatment of different cancers [135,136,137,138]. The miR-330-5p, a suppressor of oncogenesis and chemoresistance [139,140,141], can reduce S100A7 expression and subsequently inhibit the MAPK signaling pathway [68], thereby preventing EOC progression.

However, as anticancer candidates, AMPs still face many challenges and their ability to be used in vivo is doubtful. Several problems need to be solved, such as the cytotoxicity to normal cells at high concentrations, decreased activity in serum, the delivery of these molecules into target tumors and high cost of production.

## 6. AMPs and Chemoresistance

It is known that chemoresistance causes cancer relapse, dissemination and death and is an obstacle to long-term survival. AMPs are shown to be associated with chemoresistance of many tumors [142] including gynecological cancers.

AMP expression may alter the sensitivity of cancer cells to chemotherapy through diverse signaling pathways. SLPI is upregulated in human ovarian cancer cells upon exposure to paclitaxel and overexpression of SLPI is associated with paclitaxel resistance via MEK/ERK-dependent pathway [96]. Patient-derived EOC cells with high-level elafin expression show high levels of proteins previously reported to be linked to platinum chemoresistance, in particular B-cell lymphoma-extra large (Bcl-xL) and Cyclin E1 [95]. Knockdown of the elafin gene (WFDC14) increases the sensitivity of ovarian cancer cells to cisplatin, carboplatin, cyclophosphamide and 5-fluorouracil, but not paclitaxel. Particularly, caspase-3 activation and apoptosis are significantly enhanced in elafin-silenced ovarian cancer cells under cisplatin treatment [143]. S100A7-knockdown ovarian cancer cells show increased sensitivity to cisplatin although the mechanism is not clear [68].

Exogenous HBD3 is shown to protect squamous cell carcinoma of head and neck cells against cisplatin-induced apoptosis via activating the PI3K/AKT pathway [16]. In contrast, HBD3 does not protect cervical cancer cells against cisplatin- or paclitaxel-induced cell death, but instead slightly promotes cell death in vitro [12].

The expression level of AMPs in vivo is complex and seems to be closely related to the sensitivity of cancer cells to chemotherapy. However, AMPs are potential therapeutics in overcoming chemoresistance. One of the mechanisms of chemoresistance is that cancer cells pump out the corresponding chemotherapeutic drugs, whereas AMPs can avoid this mechanism by destroying the cancer cell membrane directly and rapidly. Since this mechanism is quite different from that of conventional chemotherapeutics, it seems reasonable that the combination of AMPs and conventional chemotherapeutics can synergistically enhance anticancer effect as well as reduce chemoresistance.

## 7. Concluding Remarks and Future Perspectives

The relationship between FRT-derived AMPs and gynecological cancers is complex yet intriguing. AMP expression varies by tumor and specimen type and cannot be generalized. Although the underlying mechanisms of expression changes are unknown, AMPs are potential biomarkers for detecting early cancers and predicting prognosis. Furthermore, AMP expression may act as risk factors indicative of aggressiveness of tumors, which may help determine the most beneficial treatment for the patients.

AMPs actually play a “double-edged sword” role in gynecological cancers. On one hand, AMPs have wide range of antitumor activities, they rapidly kill cancer cells, destroy primary tumors and prevent metastasis. In addition, AMPs are unlikely to induce chemoresistance due to their unique mode of action. On the other hand, AMPs are tumorigenic, pro-metastatic and closely associated to chemoresistance. This dual function of AMPs makes them potential tools or targets in clinical applications. The activities of AMPs are summarized and tabulated in Table 3. From Table 2 and Table 3, it can be seen that AMP overexpression is mainly carcinogenic, while AMP underexpression is carcinogenic or anti-tumorigenic. This result is consistent with another article based on elucidating the relationship between beta defensins and cancers [144]. However, the underlying mechanism is now unknown.

However, despite the important roles of AMPs in gynecological cancers, many uncertainties remain.

1. The relationship between the expression level of AMPs and gynecological cancers is yet to be identified.
2. There are several clinical trials of use of AMPs against infections [145]. In contrast, few clinical trials are designed for the use of AMPs on cancers [146] and none of them is related to gynecology. This may be due to the complexity of AMPs’ activities and poor understanding of the associated mechanisms.

Therefore, research on the relationship between gynecological cancers and AMPs is still in its infancy and intensive work is needed.

## Figures and Tables

**Table 1:** General information on AMPs in female reproductive tract (FRT).

AMPs	Encoded Gene	General Structures	Expression Sites in Normal FRT	Refs
HBD2 (Beta-defensin 2)	DEFB4	64 amino acids. Amphiphilic monomer. Triple-stranded, antiparallel beta sheet with strands 2 and 3 in a beta hairpin conformation.	All sites of FRT except the fallopian tubes.	[42]
HBD3 (Beta-defensin 3)	DEFB3	45 amino acids. Amphiphilic symmetrical dimer formed through strand beta2 of the beta-sheet.	Endometrium, vagina and cervix.	[43]
HNP2 (Human neutrophil peptide 2)	DEFA1	29 amino acids. An N-terminal truncated structure containing three pairs of intramolecular disulfide bond.	Cervix.	[44]
HD5 (Human α-defensin 5)	DEFA5	94 amino acids. A cationic peptide which is linked by three intra-molecular disulfide bridges, and contains six intra-molecular cysteine residues.	Endometrial, cervical and vaginal.	[45,46]
hCAP-18/LL-37 (Cathelicidin)	CAMP	37 amino acids. Amphiphilic, monomeric, α-helical peptide.	Endometrium, vagina, cervix and ovary.	[47]
SLPI (secretory leukocyte protease inhibitor)	SLPI	132 amino acids. A single-chain protein with eight intramolecular disulfide bonds.	Fallopian tube, endometrium, cervix and cervicovaginal fluid.	[48]
Elafin	PI3	117 amino acids. A structure maintained by four conserved disulfide bridges characteristic of WAP (whey acidic protein) family.	All epithelial cells lining the FRT.	[49]
HE4 (Human Epididymis Protein 4)	WFDC2	124 amino acids. A glycoprotein containing a WAP domain (4-disulfide core domain 2).	Fallopian tubes, uterus, cervix and bartholin glands.	[50]
Lysozyme	LYZ	129 amino acids. A single polypeptide.	Cervix and vagina.	[33]
Calprotectin	S100A8 S100A9	A complex of proteins S100A8 (93 amino acids) and S100A9 (113 amino acids).	Cervix and cervicovaginal fluid.	[36]
Psoriasin (S100A7)	S100A7	101 amino acids. A member of the S100 family of proteins containing 2 EF-hand calcium-binding motifs.	Vulva, vagina and ectocervix.	[51]
SP-A (surfactant protein A)	SFTPA	248 amino acids. The mature form of SP-A includes: an N-terminal segment, a collagen region, a neck region and a carbohydrate recognition domain(CRD).	Vagina.	[52]
SP-D	SFTPD	375 amino acids. Each SP-D subunit is composed of an N-terminal domain, a collagenous region, a nucleating neck region, and a C-terminal lectin domain.	Endometrium, cervix, vagina and fallopian tubes.	[53]
Lactoferrin	LTF	710 amino acids. A simple polypeptide chain folded into two symmetrical lobes (N and C lobes), which are highly homologous with one another.	Ovary, fallopian tubes and endometrium.	[54]

**Table 2:** AMP expression in gynecological cancers.

Gynecological Cancers	Expression Increased		Expression Decreased		Refs.
	Protein	mRNA	Protein	mRNA	
Epithelial ovarian cancer- tissues	HD5, hCAP-18/LL-37, SLPI, elafin, HE4, S100A7, lactoferrin	HD5, SLPI, elafin, calprotectin, HE4, S100A7, SP-D			[59,66,68,70,71,73,82,83,84,85,86,87]
Epithelial ovarian cancer- serum samples	SLPI, calprotectin, HE4				[59,61,62,63,88]
Epithelial ovarian cancer-circulating tumor cells		SP-D			[71]
Clear cell ovarian cancer- tissues	HE4	HE4			[85]
Ovarian cancer cell lines (HEY cells, SKOV-3 cells and OV-90 cells)	hCAP-18/LL-37				[87]
Ovarian cancer cell lines (Caov3 cells and SKOV3 cells)	S100A7				[68]
Cystic fluid of epithelial ovarian cancer	S100A8/S100A9				[86]
SCC (Squamous cervical cancer) --biopsies	HBD3, S100A9, S100A7		HBD2, elafin	HBD2, elafin	[12,57,58,89,90]
HSIL (high-grade squamous intraepithelial lesions)- biopsies			HBD2	HBD2	[89]
Cervical cancer cell lines (SiHa, CasKi and KT1 cells)			HBD2	HBD2	[89]
Cervical cancer cell lines (HeLa, CaSki, and SiHa cells)		HBD3	HBD3		[12]
Cervical adenocarcinoma-tissues			SLPI		[56]
Endometrioid cancer-tissues	HE4	HE4			[60]
Papillary serous endometrial cancer-tissues	HE4	HE4			[60,67]
Clear cell carcinoma of the uterus-tissues	HE4	HE4			[60]
Xenograft endometrial cancer-tissues	HE4	HE4			[91]
Endometrial carcinoma -serum samples		calprotectin			[64],
Vulvar Paget’s Disease-related vulvar cancer tissues	HE4				[76]
Vulvar squamous cell cancer-tissues	S100A7, SLPI				[65,72]
Leiomyosarcoma- serum samples	HE4				[80,81]

**Table 3:** Activities of AMPs in gynecological cancers.

AMPs	Cancers	Functions	Mechanisms	Refs.
HBD3	Cervical	Tumorigenic; pro-metastatic	Inducing cell cycle regulators and NF-κB signaling pathway.	[12]
S100A7	Cervical	Tumorigenic; pro-metastatic	Inducing ERK signaling pathway and mediating EMT.	[58]
S100A7	Ovarian	Tumorigenic; pro-metastatic; chemoresistance	Activating p38, JNK and ERK and regulating cyclin D1, MMP9 and p27.	[68]
LL-37	Ovarian	Tumorigenic, pro-metastatic (low concentrations: 0, 1, 5 and 10 μg/mL)	Activating MAPK signaling pathway and enzymes to degrade extracellular matrix.	[87,98]
LL-37	Ovarian	Anticancer (high concentrations: 50 and 100 μg/mL)	Increasing the uptake of CpG-ODN into immune cells to enhance antitumor effects.	[102]
SLPI	Ovarian	Tumorigenic; pro-metastatic; chemoresistance	Preventing cell apoptosis, inducing MMP9 and activating MAPK/ERK.	[96,97,103],
SLPI	Ovarian	Anticancer	Activating apoptosis through Caspase-2, Caspase-8 and Caspase-9.	[69]
SLPI	Endometrial	Tumorigenic	Activating cell proliferation and inhibiting growth suppressors.	[105]
HE4	Endometrial	Tumorigenic; pro-metastatic	Cell cycle control.	[91]
elafin	Ovarian	Tumorigenic and chemoresistance	Activating MAPK/ERK and NF-κB signaling pathway.	[95]
HBD-2	Cervical	Concentration-dependent: 1. 0.01–2 μg/mL, proliferation; 2.3–5 μg/mL, inhibition; 3. >20–40 μg/mL, cell lysis.	Unknown	[55]
SP-D	Ovarian	Anticancer	Inducing apoptosis.	[120]
HD5	Cervical	Reducing HPV16 infection	Directing the viral genome to the lysosome instead of trans-Golgi network.	[128]
HNP2	Cervical	Restoring normal immune function.	Inducing recruitment of dendritic cells to neoplastic lesions.	[89]
